# Definitions and pathophysiology of vasoplegic shock

**DOI:** 10.1186/s13054-018-2102-1

**Published:** 2018-07-06

**Authors:** Simon Lambden, Ben C. Creagh-Brown, Julie Hunt, Charlotte Summers, Lui G. Forni

**Affiliations:** 1 University of Cambridge, Cambridge, UK; 2 Cambridge University Hospitals NHS Foundation Trust, Cambridge, UK; 3 Surrey Perioperative Anaesthetic Critical care collaborative group (SPACeR), Intensive Care, Royal Surrey County Hospital NHS Foundation Trust, Guildford, UK; 4 Department of Clinical and Experimental Medicine, Faculty of Health and Medical Sciences, University of Surrey, Guildford, UK

**Keywords:** Vasoplegia, Shock

## Abstract

Vasoplegia is the syndrome of pathological low systemic vascular resistance, the dominant clinical feature of which is reduced blood pressure in the presence of a normal or raised cardiac output. The vasoplegic syndrome is encountered in many clinical scenarios, including septic shock, post-cardiac bypass and after surgery, burns and trauma, but despite this, uniform clinical definitions are lacking, which renders translational research in this area challenging. We discuss the role of vasoplegia in these contexts and the criteria that are used to describe it are discussed. Intrinsic processes which may drive vasoplegia, such as nitric oxide, prostanoids, endothelin-1, hydrogen sulphide and reactive oxygen species production, are reviewed and potential for therapeutic intervention explored. Extrinsic drivers, including those mediated by glucocorticoid, catecholamine and vasopressin responsiveness of the blood vessels, are also discussed. The optimum balance between maintaining adequate systemic vascular resistance against the potentially deleterious effects of treatment with catecholamines is as yet unclear, but development of novel vasoactive agents may facilitate greater understanding of the role of the differing pathways in the development of vasoplegia. In turn, this may provide insights into the best way to care for patients with this common, multifactorial condition.

## Background

**Fig. 1:**
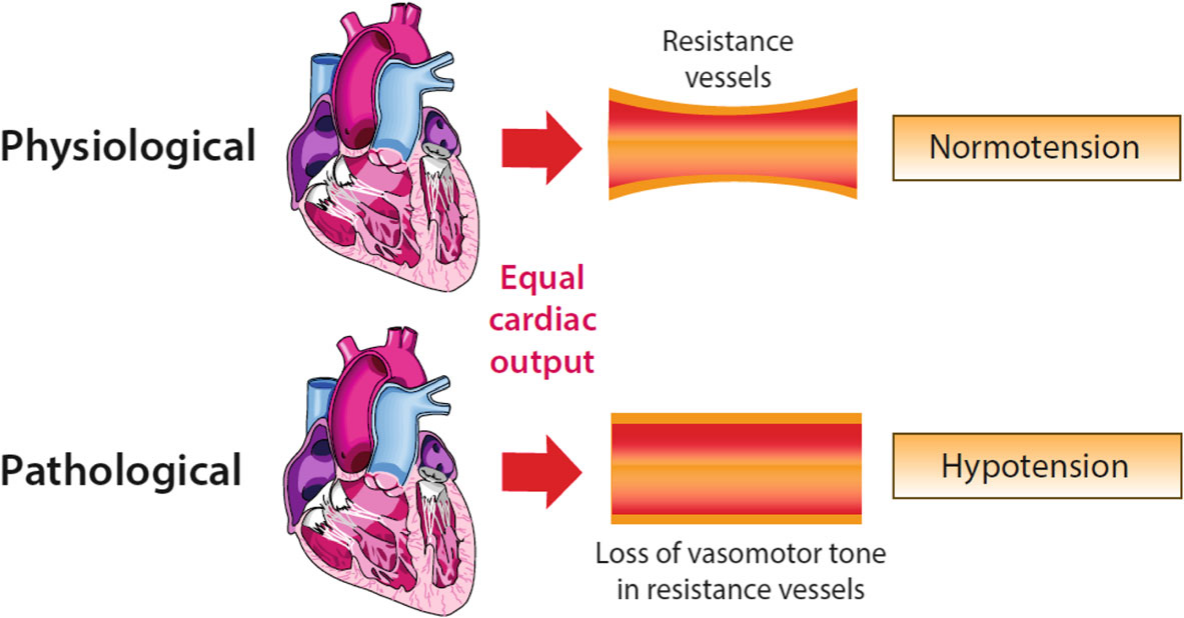
The relationship between tone in resistance vessels, under conditions of equal cardiac output, and the systemic blood pressure—preserved vasomotor tone leading to normotension and loss of vasomotor tone leading to hypotension

Vasoplegia is an abnormally low systemic vascular resistance (SVR) that is manifest as profound hypotension or the requirement for therapies to avoid this, in the presence of a normal or increased cardiac output (Fig. 1). Physiologically, a low SVR is defined as a low ratio of difference in blood pressure between arterial (MAP) and venous pressures (RAP) to the cardiac output [SVR = (MAP − RAP)/CO]. Clinically, vasoplegia is often recognised in the absence of such comprehensive haemodynamic data. The causes of vasoplegia are diverse, and several definitions have been described for specific causes; similarly, related terminologies are variably used. The absence of consensus clinically based definitions of vasoplegia impede progress in understanding the pathophysiology of vasoplegia; this is particularly true when considering the similarities between vasodilatory shock due to sterile or non-sterile causes—for example hypotension despite adequate fluid resuscitation in early burns injury versus early sepsis.

**Fig. 2:**
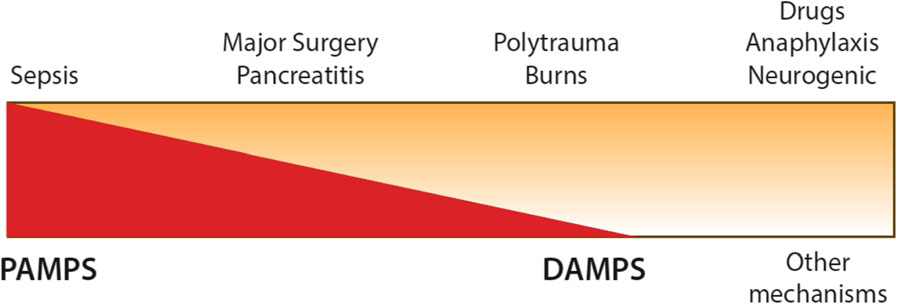
The main clinical causes of vasoplegia (*top*) and how they are perceived to relate to underlying aetiologies (*bottom*)—i.e. sepsis is predominantly a response to PAMPS (pathogen-associated molecular patterns) compared to burns or polytrauma where DAMPS (damage-associated molecular patterns) are the major cause

Patients in hospitals most commonly experience hypotension due to vasodilatation because of the administration of general or neuraxial anaesthesia, and even when transient this has been associated with adverse outcomes [1–3]; however, further discussion is beyond the scope of this review. Similarly, hypotension due to vasodilatation resulting from neurogenic shock has a discrete pathophysiology (loss of sympathetic innervation due to spinal cord injury) and is not considered further. This review focuses on the causes of vasoplegia that reflect a varied response to pathogen-associated molecular patterns (PAMPS) and damage-associated molecular patterns (DAMPS) (Fig. 2). The response to these stimuli generates a combination of vasodilatation and increased capillary permeability. Capillary leak, coupled with greater vessel capacitance mediated by vasoplegia may result in absolute, or more commonly relative, hypovolemia. Fluid resuscitation to treat this phenomenon is a standard of care, but this does not treat the underlying pathology and positive fluid balance is associated with harm [4].

It is uncertain if it is justifiable to consider vasoplegia to be a pathophysiologically distinct entity representing uncontrolled failure of vascular homeostasis or to represent the end of a spectrum of vasodilatation.

Vasoplegic shock (VS), synonymous with distributive shock, is a more significant circulatory perturbation that is best described as vasoplegia with evidence of tissue hypoperfusion which may be accompanied with hyperlactataemia [5]. The presence of a raised lactate portends a particularly grave prognosis in the presence of shock or indeed septic shock [6, 7]. This review describes the key mechanisms involved in the development of VS, a process that is mediated by a diverse set of pathways which combine and contribute to the evolution of the shock state. Advancing our understanding of these pathways and their role in the transition from adaptive physiological to maladaptive pathological response may provide novel diagnostic tools, prognostic insights and therapeutic targets to guide the management of vasoplegia.

To date, our treatment options are limited and do not target some of the main pathophysiological pathways. First-line vasopressor therapy is typically with catecholamines and resistance is referred to as catecholamine-resistant hypotension (CRH). Although vasopressor infusion is required in order to maintain an adequate MAP, significant variation remains in clinical practice, particularly with regard to personalised targets depending on premorbid characteristics, and current research efforts are addressing this issue [8]. Moreover, it is well recognised that infused catecholamines are associated with a range of adverse effects on the metabolic, immune and coagulation systems [9, 10].

The tools available to clinicians to monitor the severity and impact of vasoplegia are limited [11, 12] and existing treatment goals may not result in the desired tissue level effects on microvascular flow [13]. Improved understanding of the pathophysiology of vasoplegia combined with new tools to monitor the impact of interventions on vessel function may lead to the development of the next generation of vasoactive therapies. The measurement of cardiac output, systemic blood pressure and central venous pressure allow derivation of the SVR, although targeting ‘normal’ values with insufficient consideration of their components may be hazardous [13].

## Causes of vasoplegia

### Sepsis

The commonest cause of vasoplegia in critical care is sepsis. The incidence is dependent upon the definition used and the patient population under consideration [14]. Receipt of vasopressors, where appropriate, is now recognised as a cardinal feature of septic shock and indeed the most recent definition of septic shock does not require the presence of persistent hypotension. However, it includes administration of vasopressors to maintain a mean arterial pressure (MAP) of 65 mmHg (in the absence of hypovolaemia) and an elevated blood lactate level [15] in the presence of sepsis. This contrasts with earlier definitions which required hypotension as reflected by a low MAP (< 60 mmHg) in the absence of hypovolaemia and other cause of hypotension [16].

### Cardiac surgery

Vasoplegia and VS occurring in patients following cardiac surgery are the second commonest cause. Diagnosis is more complex as there is an ever-present risk of impaired cardiac output contributing to hypotension, the cause of which must be ascertained early—differentiating between reduced preload from bleeding, impaired myocardial contractility or the occurrence of cardiac tamponade. The second main complicating factor is the frequent use of vasodilatory inotropes that directly influence vascular tone. Therefore, although no consensus definition exists, there are several working definitions that combine i) hypotension in the absence of a low cardiac output state and ii) absence of infection; additional criteria may also include the absence of vasodilatory inotropes such as dobutamine or milrinone, or presence of evidence of tissue hypoperfusion. Clinical factors that predispose to the development of vasoplegia following cardiac surgery have been described [17–19] and various treatment regimens considered, including the use of alternative vasoconstrictors [20–22]. Although vasoplegia following cardiac surgery is often attributed to exposure to an extracorporeal circuit the evidence in support of this remains mixed [23].

### Non-cardiac surgery

Hypotension due to vasodilatation in patients following major non-cardiac surgery is usually manifest as requirement for vasopressors to maintain an adequate MAP following appropriate resuscitation to restore euvolaemia, and its incidence is seldom reported. Reported risk factors include prolonged surgery and significant requirement for blood transfusion [24, 25]. Where postoperative admission to a critical care environment is routine, the use of vasopressors in the postoperative period to support blood pressure following optimisation of fluid status is commonplace. Although vasopressors may be required to counteract the systemic vasodilatory effects of neuraxial blockade, such as epidural analgesia, where requirements are significant in an adequately resuscitated patient then this should be considered to be vasoplegia.

#### Burns, trauma and pancreatitis

These are conditions united by significant tissue injury, with consequent hypermetabolism, systemic inflammation and predisposition to developing organ dysfunction. Vasoplegia could be considered to be one such organ dysfunction, and is a recognised complication of polytrauma, burns [26–28] and, even in the absence of infection, severe pancreatitis—where vasoplegia is associated with adverse outcome [29, 30].

## The pathophysiology of vasoplegia

### Normal physiology

SVR is determined by changes in arteriolar diameter, controlled by the contractile activity of the vascular smooth muscle cells (VSMC) in the tunica media. The contractile state of the VSMC is referred to as the vascular ‘tone’ and is regulated through intracellular calcium (Ca^2+^) concentration. VSMC contraction is driven by a rise in cytosolic Ca^2+^ concentration through release of stored Ca^2+^ from the sarcoplasmic reticulum as well as extracellular Ca^2+^ influx through voltage-sensitive channels. Relaxation of the VSMC is driven by a fall in cytosolic Ca^2+^, due to uptake of Ca^2+^ by the sarcoplasmic reticulum and expulsion of potassium (K^+^) or Ca^2+^ (via K^+^ channels and Ca^2+^-ATPase pumps) into the extracellular space, resulting in cellular hyperpolarisation and vasodilation. Vascular tone is therefore dependent on the rate of Ca^2+^ influx versus removal, which in turn is regulated by intrinsic and extrinsic mechanisms [31]. Intrinsic regulators include:

i. endothelial secretions (nitric oxide, prostacyclin, endothelin)
ii. vasoactive metabolites (acidosis, hypoxia, hydrogen peroxide)
iii. autacoids (serotonin, prostaglandins, thromboxane A_2_)

Extrinsic regulation is largely mediated by sympathetic neural control and vasoactive hormones, which include adrenaline, angiotensin II and vasopressin.

### The pathophysiology of vasoplegia: intrinsic regulators

#### Nitric oxide

Nitric oxide (NO), first identified as the endothelial-derived relaxing factor (EDRF) [32], is a critical regulator of vascular function in both health and disease. NO diffuses freely from the endothelium into the neighbouring VSMC and bloodstream causing vasodilation, inhibition of VSMC proliferation, platelet activation and leukocyte adhesion. It is generated from L-arginine by endothelial nitric oxide synthase [33], and to a lesser extent neuronal nitric oxide synthase [34] (eNOS and nNOS, respectively). These calcium-dependent constitutive isoforms produce NO in picomolar concentrations and this induces cGMP-PKG-mediated vasodilation [35–37]. Inflammatory autacoids, including bradykinin and thrombin, increase NO production and vasodilation by activating eNOS. In addition, inflammatory cytokines and PAMPs such as lipopolysaccharide (LPS) induce the synthesis of the third calcium-independent, inducible NOS isoform (iNOS). This results in an increase in NO of two to three orders of magnitude above baseline and is a major driver of acute vascular dysfunction in shock [38]. Administration of non-selective inhibitors of NOS has been shown to be associated with improvement in haemodynamics in patients with septic shock but also, despite this, increased mortality—probably through the impact of NOS inhibition on immune cell and cardiac NO production [39, 40]. Therapies that target the vasculature and regulate, but not entirely abolish, the increase in NO synthesis may offer a more favourable profile to those previously tested to-date in clinical trials [41].

#### Prostanoids

Prostacyclin (PGI_2_) is produced by the endothelium constitutively and causes platelet aggregation [42] and induces cAMP-PKA-mediated vasodilation [43, 44]. Prostacyclin production is greatly increased in inflammation and contributes to vasodilation. A broad range of inflammatory stressors and/or PAMPs, including interleukin 1(IL-1), tumour necrosis factor α (TNF- α), hypoxia and LPS, provoke the induction of COX-2 isoform and increased synthesis of PGI_2_ by prostacyclin synthase (PGIS) [45–47], which drives vasoplegia. Therapeutic trials of nonselective COX inhibition in sepsis proved inconclusive, with any beneficial effects on the degree of vasoplegia mediated by PGI_2_ likely offset by other prostaglandin-mediated actions [48].

A short lived prostainoid, thromboxane A2 (TXA_2_) opposes the actions of PGI_2_ and promotes vasoconstriction and platelet aggregation [49]. Therefore TXA_2_ has been implicated as a potential causative factor in the increased risk of cardiac ischaemia in patients taking COX2 inhibitors [50]. TXA_2_ regulates vascular tone through binding to thromboxane-prostanoid (TP) receptors in vascular smooth muscle and, in keeping with other agents, promotes calcium influx and vascoconstriction [51]. Animal studies have suggested that knockout of the TP receptor is associated with reduced iNOS expression and protection against vascular hyporesponsiveness, suggesting a role for TXA_2_ as a regulator of vasoplegia [52, 53]. In humans, limited evidence suggests that the balance between TXA_2_ and PGI_2_ may be important with high relative levels of TXA_2_ associated with worse outcome in a preliminary study of patients with sepsis [54].

#### Endothelin 1

Endothelin 1 (ET1) is the predominant isoform of the endothelin family and is a small peptide which acts as a vasoconstrictor [55]. ET1 activates endothelin A (ETA) receptors in the VSMC, which again drive the elevation of intracellular Ca^2+^ and contraction [56]. Subtypes of endothelin B (ETB) receptors, found in the endothelium and vascular smooth muscle, act as an autoregulatory mechanism for controlling basal tone through vasodilatation and smooth muscle contraction [57]. In conditions of inflammatory stress, however, ET1 has potentially deleterious effects through the activation of a number of signalling pathways, increasing synthesis of IL-1, TNF-a and IL-6 [58]. Selective and non-selective blockade of the ET receptor subtypes have been shown to have promise in a range of animal models [59].

#### Oxygen free radicals

Uncoupling of endothelial NOS enzymes may cause an increase in reactive oxygen species and mitochondrial dysfunction [33]. The superoxide anion may reduce NO to form peroxynitrite (ONOO^−^), which acts as a powerful oxidising agent that provokes cellular dysfunction and vasoplegia [60]. Under physiological conditions, the superoxide radical anion is metabolised by superoxide dismutase (SOD). Non-enzymatic mechanisms for superoxide metabolism are mediated by ascorbic acid and uric acid. In shock states, excess NO production results in excess ONOO^−^ production, which may be attenuated by antioxidants [61], and reactive oxygen species (ROS) may also cause the deactivation of catecholamines, a phenomenon that can be reversed by the administration of a synthetic mimic of superoxide dismutase [62].

#### Hydrogen sulphide

Hydrogen sulphide (H_2_S) is synthesised from the amino acid L-cysteine through vitamin B6-dependent cystathionine-β-synthase or cystathionine-γ-lyase [63]. H_2_S readily diffuses into the vascular smooth muscle and at low concentrations may have cytoprotective effects, although in sepsis concentrations are significantly elevated [64]. At higher concentrations, H_2_S contributes to the development of vasodilatory shock through a range of oxygen-dependent actions, including inhibition of cytochrome c oxidase with impairment of mitochondrial function, activation of potassium ATP channels and inhibition of endothelial angiotensin converting enzyme activity [63, 65–67]. In addition, H_2_S interacts with NO, which may attenuate NO actions [68, 69]. H_2_S has also been suggested as a potential therapeutic agent leading to the development of a cytoprotective hibernation-like state. Animals treated with H_2_S are protected from both lethal hypoxia [70] and haemorrhage [71]. This finding has led to the pre-clinical study of H_2_S treatment in modulating the deleterious effects of ischaemia-reperfusion injury in experimental models, including porcine myocardial injury [72].

#### Non-endothelial: potassium channel hyperpolarisation

As indicated, efflux of potassium through ATP-sensitive potassium channels is an important mechanism for the regulation of VSMC membrane potential. Over-activation of potassium channels results in hyperpolarization of the cell, with resulting inactivation of voltage-gated calcium channels. The subsequent vasodilatation is an important driver of vascular dysfunction. In addition to endothelial-derived mediators, a number of circulating factors can drive potassium channel-mediated vascular dysfunction, including hypoxia [73], reduced pH [74] and increased circulating lactate [75]. The vascular dysfunction induced by inflammatory stress such as endotoxin [76] led to the hypothesis that inhibition of potassium channels may offer a novel therapeutic strategy. Animal models showed haemodynamic improvements following inhibition with the specific ATP-sensitive potassium channel blocker glibenclamide [77]. However, phase 2 randomised controlled trials in human subjects demonstrated no benefit [78], and concerns regarding non-vascular effects limit its potential utility [79].

### The pathophysiology of vasoplegia: extrinsic regulators

#### Catecholamine resistance

The development of vasoplegia may also be driven by changes in the efficacy of circulating catecholamines in generating VSMC contraction. Animal models suggest that in later stages of sepsis, alpha-1 adrenoceptor expression falls, resulting in peripheral resistance to norepinephrine [80, 81]. In human studies, the expression of peripheral receptors appears to be related to illness severity, with increased expression in mild disease and reduced expression observed in severe sepsis, suggesting that in patients with vasoplegia, a similar pattern to that observed in rodent models may occur [82].

#### Corticosteroid response

Glucocorticoids drive diverse tissue responses in inflammation, including circulating immune cell function and cytokine release [83]. These processes are driven by regulation of a number of intermediate pathways, including inducible NOS-mediated NO synthesis and COX2 activity [84]. In the vasculature, steroid receptors are present in both endothelial and vascular smooth muscle and, under physiological conditions, potentiate the response to circulating catecholamines and angiotensin II [85, 86]. In addition, the rapid cellular actions of steroids can promote increased concentrations of second messengers such as inositol-3-phosphate and cAMP [87, 88]. Limited evidence suggests that critical illness-related corticosteroid insufficiency may develop in shock states. Causes of this insufficiency include relative insufficiency of the HPA axis [89], adrenal failure [90] or necrosis [91], and in some cases peripheral resistance to corticosteroids [90]. These factors may combine to exacerbate vascular dysfunction in shock and provide a mechanism for the proposed benefit of exogenous steroid administration to reduce the severity or duration of vasopressor dependence in septic shock [92–94].

#### Endogenous vasopressin

Vasopressin acts via specific V1 receptors on the VSMC surface to promote increased intracellular calcium via G protein-coupled receptors and phospholipase C, which in turn drives contraction. In septic shock, plasma concentrations of vasopressin increase in the early stages of shock; however, after 24 h levels fall to sub-normal levels, which may be a mechanism for loss of vascular tone [95]. This may be associated with a reduction in peripheral receptor numbers, a phenomenon observed in animal models [81]. In addition, V2 receptors on endothelial cells may provoke vasodilatation via the increased synthesis of NO [96].

## Conclusions

**Fig. 3:**
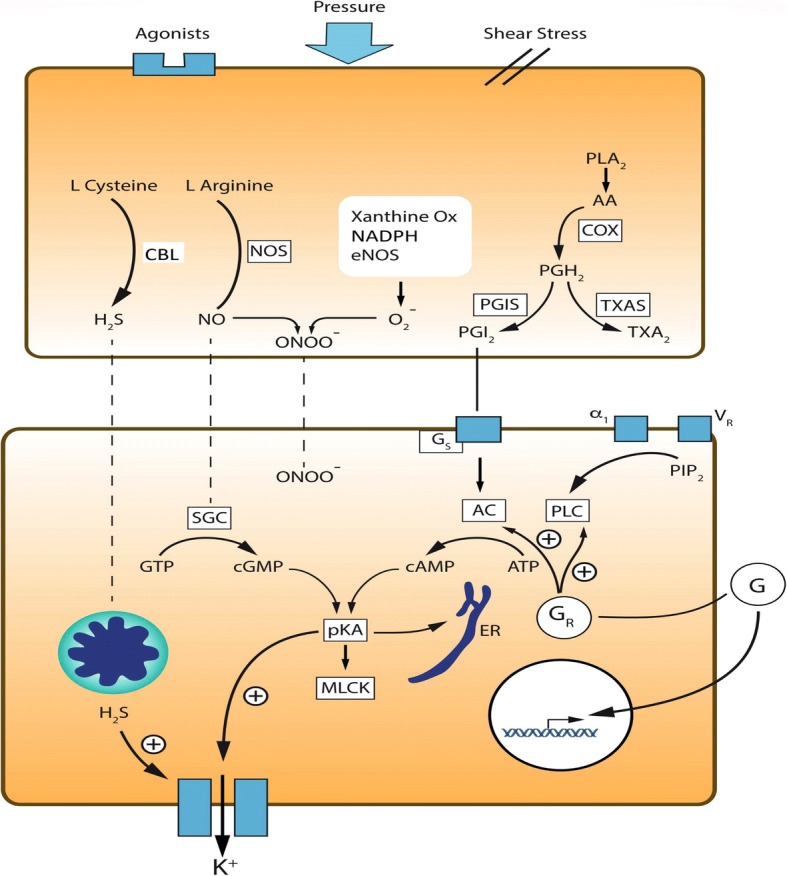
Endothelial and smooth muscle-mediated mechanisms of vascular dysfunction in shock. Hormonal and mechanical factors drive endothelial cell activation in the vasculature. Increased expression of the inducible isoform of nitric oxide synthase (*iNOS*) generates increased production of nitric oxide (*NO*) from arginine. NO directly reduces vascular tone through the activation of soluble guanylate cyclase, which catalyses the conversion of GTP to cyclic GMP. In addition, NO combines with oxygen free radicals (O_2_^−^) produced by dyfunctional mitochondria and a number of enzymes, including endothelial nitric oxide synthase (*eNOS*), NADPH and xanthine oxidase. The synthesised peroxynitrite also directly contributes to smooth muscle relaxation. Hydrogen sulphide (*H*_*2*_*S*) is synthesised from L-cysteine by cystathionine-β-synthase or cystathionine-γ-lyase (*CBL*). In shock, H_2_S reduces vascular tone through inhibition of mitochondrial function and activation of potassium channels. Arachidonic acid is converted to vasoactive prostaglandins via a two-step pathway involving cyclooxygenase (*COX*) isoforms and prostacyclin synthase (*PGIS*), which synthesises prostacyclin (*PGI*_*2*_). This in turn drives vasodilatation via the activation of stimulatory G-protein-coupled receptors (*Gs*), which promotes synthesis of cyclic AMP (*AMP*) from ATP by adenylate cyclase (*AC*). Thrombxane A2 (*TXA2*) is synthesised from the common intermediate PGH_2_ and plays a role in the regulation of vascular tone in shock states. In the smooth muscle, activation of protein kinase A (*PKA*) by a number of routes drives smooth muscle relaxation through potassium channel- and endoplasmic reticulum (*ER*)-mediated hyperpolarization and activation of myosin light chain kinase (*MLCK*). Glucogorticoids (*G*) activate glucocorticoid receptors (*GR*) through both classic and non-classic mechanisms to regulate vascular tone, a process that is impaired in a number of ways in shock. Changes in expression of adrenergic (*α*_*1*_) and vasopressin (*V*_*R*_) receptors and their circulating agonists impair the function of vascular smooth muscle in shock states

Although vasoplegia is a well-recognised phenomenon, it still suffers from the lack of a unifying clinical definition. This prevents clinical trialists and translational scientists from sharing the common language necessary to facilitate research and increase understanding of this phenomenon. Certainly, we believe that a uniform approach to describing vasoplegia would reap benefits and stimulate further investigation of the underlying pathophysiological mechanisms. Vasoplegia is a complex phenomenon centred around vascular reactivity with multiple contributory potential mechanisms (outlined in Fig. 3). The advent of further alternatives to catecholamines, such as angiotensin II [97], may herald a new approach to treatment and the potential for alternative approaches—for further details, the reader is invited to consult the treatment article published in the same series. Optimum targets for systemic blood pressure remain contentious, and increasingly and appropriately, the pharmacological agents used to achieve these goals will be more closely scrutinised.

## Abbreviations

CO: Cardiac output
CRH: Catecholamine-resistant hypotension
DAMPS: Damage-associated molecular patterns
MAP: Mean arterial pressure
MLCK: Myosin light chain kinase
PAMPS: Pathogen-associated molecular patterns
RAP: Right atrial pressure
ROS: Reactive oxygen species
SVR: Systemic vascular resistance
VS: Vasoplegic shock
VSMC: Vascular smooth muscle cells
